# Supporting schools during the implementation of the health-promoting school approach: The roles of a healthy school advisor

**DOI:** 10.3389/fpubh.2022.960873

**Published:** 2022-12-15

**Authors:** Nina Bartelink, Bonnie van Dongen, Stef Kremers, Carry Renders, Boukje van Vlokhoven, Marije van Koperen, Patricia van Assema

**Affiliations:** ^1^Department of Health Promotion, Care and Public Health Research Institute (CAPHRI), Maastricht University, Maastricht, Netherlands; ^2^Department of Health Promotion, School of Nutrition and Translational Research in Metabolism (NUTRIM), Maastricht University, Maastricht, Netherlands; ^3^School of Sport Studies, Fontys University of Applied Sciences, Eindhoven, Netherlands; ^4^Department of Health Sciences, Faculty of Science, Amsterdam Public Health Research Institute, Vrije Universiteit Amsterdam, Amsterdam, Netherlands; ^5^Sector Organization for Secondary Education (VO-raad), Utrecht, Netherlands; ^6^Programma Gezonde School (Healthy School Program), Utrecht, Netherlands; ^7^Department of Public Health and Health Services, National Institute for Public Health and the Environment (RIVM), Bilthoven, Netherlands

**Keywords:** health-promoting school approach, healthy school advisor, context-oriented implementation, co-creation, professional development

## Abstract

**Introduction:**

The health-promoting school (HPS) approach was developed by the World Health Organization to create health promotion changes in the whole school system. Implementing the approach can be challenging for schools because schools are dynamic organizations with each a unique context. Many countries worldwide have a health promotion system in place in which healthy school (HS) advisors support schools in the process of implementing the HPS approach. Even though these HS advisors can take on various roles to provide support in an adaptive and context-oriented manner, these roles have not yet been described. The current study aims to identify and describe the key roles of the HS advisor when supporting schools during the dynamic process of implementing the HPS approach.

**Methods:**

The study was part of a project in which a capacity-building module was developed for and with HS advisors in the Netherlands. In the current study, a co-creation process enabled by participatory research was used in which researchers, HS advisors, national representatives, and coordinators of the Dutch HS program participated. Co-creation processes took place between October 2020 and November 2021 and consisted of four phases: (1) a narrative review of the literature, (2) interviews, (3) focus groups, and (4) a final check.

**Results:**

Five roles were identified. The role of “navigator” as a more central one and four other roles: “linking pin,” “expert in the field,” “critical friend,” and “ambassador of the HPS approach.” The (final) description of the five roles was recognizable for the HS advisors that participated in the study, and they indicated that it provided a comprehensive overview of the work of an HS advisor in the Netherlands.

**Discussion:**

The roles can provide guidance to all Dutch HS advisors and the regional public health organizations that employ them on what is needed to provide sufficient and context-oriented support to schools. These roles can inspire and guide people from other countries to adapt the roles to their own national context.

## Introduction

Schools can contribute to the promotion of health and wellbeing among children and adolescents since a significant proportion of a child's day is spent there and schools have the ability to reach all children from a variety of backgrounds (1). However, health promotion (HP) is often not part of the school's educational goals, as the school's main responsibility is teaching. To bring together the sectors of education and health and promote health and wellbeing for all stakeholders (including pupils) in the school, the health-promoting school (HPS) approach was developed by the World Health Organization (WHO) in the late 1980's (2). With this approach, the WHO advocated for a whole-school approach that not only focuses on health education in the classroom but also on creating a healthy school (HS) environment, HS policies, and attention to HP in the whole curriculum. In other words, creating change by embedding HP in the whole school system. However, implementing a system-wide change is challenging because schools are dynamic organizations in which components, people, and the environment are continuously interacting, adapting, and changing (3–6). A consequence of these continuously changing conditions is that each school is unique and a school always operates in its own specific context. A school context is defined here as *the specific circumstances and characteristics of a school, which relates to the social, political, economic, and physical environment; the characteristics, behaviors, wishes, and needs of the people in the school; the wider community in which the school is located; as well as the history and organization of the school* (7, 8). A unique school context thus means that each school has its own specific needs, wishes, and opportunities, and flexibility is needed to be able to deal with each unique context. Consequently, a tailored translation of the HPS approach to this unique school context is required in each school to create effective and sustainable HP actions and activities.

Creating such a tailored translation of the HPS approach is not an easy task for the stakeholders in a school. Therefore, a school can benefit from getting support from an HS advisor who can help them to translate, develop, implement, and evaluate HP actions and activities that fit the school's context. HS advisors are seen here as (mostly) external and local health (promotion) professionals. Such an advisor has previously been described by Boot et al. as a professional that can be seen as an important change agent who convinces the school of the benefits of working on HP and who guides the process of change (9, 10). Many countries worldwide have a health promotion system in place in which HS advisors support schools in the process of implementing the HPS approach.

Several studies have described various tasks of HS advisors in their support to schools, such as being a contact person who maintains links between a school and local HP partners or an advisor with knowledge on health promotion interventions (11, 12). As such, tasks can be seen as something that must be fulfilled by an HS advisor. Currently, specific tasks of HS advisors are not structurally established within the HPS approach. Moreover, tasks to provide optimal support may vary constantly due to the unique and dynamic context of a school (3, 5). It, therefore, might be beneficial to explore the overarching roles of HS advisors instead of specific tasks. By defining several roles, a potential frame of reference can be established for HS advisors to optimally support schools throughout the implementation process of the HPS. Roles, comprised of a variety of tasks, offer more flexibility to HS advisors to adjust support to the dynamic and unique situation of a school, and support them in an adaptive and context-oriented manner. Therefore, this article explores the following research question: *What are the key roles of HS advisors when supporting schools in the implementation process of the HPS approach?* This study will focus on the Dutch context because a translated version of the HPS approach already runs for several years and also includes the support of an HS advisor.

## Materials and methods

### The HS program in the Netherlands

The implementation of the HPS approach in the Netherlands finds its origin in a project called SchoolBeat (“SchoolSlag”) (12). Since SchoolBeat, new developments in the implementation of the HPS approach in Dutch schools have taken place, and schools have increasingly been approached as whole systems (11, 13). Eventually, this has led to the national “Healthy School program.” This program aims to integrate HP into the DNA of every school in the Netherlands. The program is funded by the Dutch government and is nationally coordinated by the Association of Public Health Services (GGD GHOR Nederland), the National Institute for Public Health and the Environment (RIVM), and the primary, secondary, and vocational education councils (in Dutch: PO-Raad, VO-Raad, and MBO Raad). The program covers 10 health topics: (1) nutrition, (2) exercise and sport, (3) preventing smoking, alcohol abuse, and drug abuse, (4) wellbeing, (5) hygiene, (6) media literacy, (7) hearing (loss), (8) sleep, (9) relationships and sexuality, and 10) environment and nature. Schools can decide which health topic(s) to focus on. To create change in the whole school system, schools are stimulated to focus on four pillars per health topic: (1) health education, (2) a healthy social and physical environment, (3) HS policy, and (4) signaling specific health or health behavior issues among children (that warrant special attention or care of other health professionals). Schools can earn a topic-specific HS certificate if they adhere to specified quality criteria, which are based on the four pillars. Moreover, as part of the program, it is advocated to appoint a school employee as an HS coordinator within the school. This HS coordinator is the central contact person in the school for HP and coordinates the implementation of all actions and activities in the school related to HP, thereby informing and involving all stakeholders in the school. Finally, as part of the program, schools can apply for support for its implementation. This support consists of training for the HS coordinator on the HS program, 10 h of advice from an HS advisor, and an additional 3,000 EURO to spend on HP activities or to reimburse the HS coordinator's working hours. In the Netherlands, the HS advisor is employed at a regional Public Health Service. There are 25 of these Public Health Services in the Netherlands, each servicing multiple municipalities^1^.

### Study design

A participatory research approach was used in the current study by focusing on a process of sequential reflection and action, carried out together with HS advisors (14). The study is part of a Dutch research project in which a capacity building module is developed for and with HS advisors. The goal of this module is to create awareness among HS advisors of the various roles they can have and improve their ability in supporting schools in a more adaptive and context-oriented manner. Ethical approval for this project was obtained from the Faculty of Health, Medicine and Life Sciences Research Ethics Committee from Maastricht University, The Netherlands (FHML-REC/2021/001). The project was based on a co-creation process, which can be defined as a process in which new knowledge is generated via the honest, democratic, and meaningful engagement of key cross-sectoral stakeholders, making it sensitive to context and more likely to be implemented (15, 16). The focus of this article is on the parts of the process that are relevant to the current study.

### Participants

**Figure 1:**
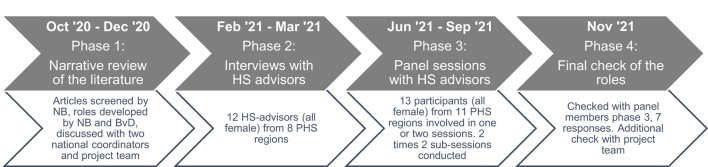
Phases of the current study.

Two principal researchers (NB and BvD) facilitated the co-creation process. Participants who contributed to this study were HS advisors employed at different regional Public Health Services in the Netherlands, two national coordinators of the HS advisors, and a project team. This project team consisted of two national representatives of the HS program, as well as the two principal researchers and three other researchers in the field of school health promotion. All participants provided feedback on the base of their experience, knowledge, and expertise. The co-creation processes relevant to the current study took place from October 2020 to November 2021 and consisted of four main phases (Figure 1): (1) a narrative review of the literature, (2) individual interviews with HS advisors, (3) focus groups with HS advisors, and (4) a final check of the roles. In order to enable a co-creation process that builds on the results of each phase, insights were discussed with the project team at the end of each phase. Moreover, when applicable, the results were also discussed with two national coordinators of the HS advisors.

### The four phases in the co-creation process

#### Phase 1: A narrative review of the literature

A narrative review of the scientific literature was performed to explore what was already known worldwide about the supporting role of an HS advisor in schools during the implementation process of the HPS approach. A literature search was conducted by NB from October 2020 to November 2020 with a focus on identifying tasks that HS advisors performed or are stimulated to perform. Both principal researchers (NB and BvD) had extensive knowledge regarding the existing literature on the implementation of the HPS approach. Based on this expertise, the first selection of articles was made in which the focus was on the implementation process of the HPS approach. Then, the snowballing method was used in which the reference list of the articles was checked to identify other possible relevant articles. Finally, all articles were screened to identify the ones that describe (potential) tasks of HS advisors. When this was the case, the article was included. All tasks were extracted from the articles. The extracted list of tasks was grouped by NB, as overlap existed among them. Overarching these tasks, several key roles were identified. These key roles were described based on the tasks found in the articles. The identified roles and its description were first discussed with the other principal researcher (BvD). Following this, they were discussed with the two national coordinators of the HS advisors. Finally, the roles were discussed in the project team.

#### Phase 2: Individual interviews with HS advisors

Individual semi-structured interviews with Dutch HS advisors were conducted to gain insight into their experiences and perceptions regarding the support they provide to schools during the implementation process of the HS program. In addition, identified roles of an HS advisor and their descriptions were introduced to check and improve these roles. The interviews were held online from February 2021 to March 2021 by one of the two principal researchers. The participants of the interviews were recruited by using the existing network of the two researchers and in consultation with the involved representatives of the HS program. Two inclusion criteria were used: (1) no more than two HS advisors from the same regional public health services to ensure that the participants were as representative as possible for all the HS advisors in the Netherlands and (2) sufficient practical experience with supporting schools in the HS program. Therefore, HS advisors who had less than a year of experience were excluded. The number of included participants was based on data saturation. All participants were recruited via email with information about the aim of the study and the procedure and duration of the interview. All participants signed an informed consent form before the interview.

The interviews were based on an interview guide consisting of two parts. The first part focused on their HS advisory work in general to gain a deeper understanding of their daily tasks and roles. This was done in order to let the HS advisor speak freely about their roles and tasks without framing from the roles identified in the narrative review phase. Questions were divided into three implementation phases of the Dutch HS program, such as (1) (adoption and) preparation in which an HS advisor is getting acquainted with a school, (2) execution in which a school is actively working on HS activities supported by an HS advisor, and (3) continuation in which an HS advisor is working with a school for several years. For each phase, the following questions were asked: *How do you do your work in general? What are the most important tasks? What do you need to be able to support a school in this phase? To what extent do you adapt what you do to the specific situation of a school?* The second part of the interview focused on the results of the narrative review. We asked whether the results of the narrative review, i.e., the identified roles and their descriptions, were recognizable, if they provided a complete overview of their work and their various roles in relation to the school, and if the HS advisor had any suggestions for improvements or adjustments. All interviews were audio-recorded, and a summary was written after each interview and sent to the participant for member checking. Based on these recordings and summaries, the two principal researchers applied a thematic analysis technique, beginning with open coding to identify the tasks and competencies mentioned by HS advisors. Then axial and selective coding was applied to group these findings into the roles identified in the narrative review. This was done to provide better insight into these roles and/or readjust them, and to develop additional roles if HS advisors had mentioned tasks that did not fit in these roles. The overall findings and insights were fed back and discussed with the project team.

#### Phase 3: Focus groups with HS advisors

The insights from phase 2 were used to further improve the identified roles and their descriptions. The roles were then discussed in several focus groups with HS advisors to see whether the improvements were helpful and to investigate whether adjustments were needed. All focus groups followed a semi-structured setup with several topics to discuss based on the development of the training module for HS advisors. Group discussions among participating HS advisors were encouraged. Participants were recruited in April and May 2021 via the snowballing method. First, the HS advisors who participated in the interviews were asked to join the focus groups. Then, these HS advisors were asked whether they had colleagues or knew other HS advisors who would like to join. Participants could sign up if they were interested and decide per focus group session whether they would join that specific session. Financial compensation was offered for every hour they participated. Two online focus groups were held, the first in June 2021 and the second in September 2021. In order to keep the group size manageable for online sessions, in a way that everyone had the opportunity to fully contribute, both sessions were divided into two sub-sessions (sub-sessions 1a and 1b, and sub-sessions 2a and 2b). Even though the content of these focus groups was mainly related to the development of the module, the roles were also part of the discussions during these sessions. In the June sessions (1a and 1b), the roles and their descriptions were presented and discussed in the group. The participants were hereby asked, similar to the interviews, whether the roles were recognizable, whether they provide a complete overview of their work and their various roles in relation to the school, and whether they had suggestions for improvements or adjustments. In the September sessions (2a and 2b), the participants were asked to apply for the roles by discussing a real-life situation in the daily work of an HS advisor (the case discussed was: no support among the school team members for the implementation of the HPS approach). Each sub-session was recorded, transcribed, and summarized. The summary was sent to the participants of that specific sub-session for member checking. Based on the transcriptions and summaries, the two principal researchers conducted a thematic analysis to identify perceptions of HS advisors of their role and tasks in general, as well as the roles developed in previous phases. The two researchers discussed findings and insights in order to explore whether the roles were complete and/or needed readjustments. These overall findings were combined into two overarching summaries: one summary for the June sessions (1a and 1b) and the other summary for the September sessions (2a and 2b). These summaries were fed back and discussed with the project team.

#### Phase 4: Final check of the roles

The insights from phase 3 were used to make any last improvements and finalize the identified roles and their descriptions. Also, the roles were visualized in a model to show how they relate to each other. In November 2021, the roles, their descriptions, and the model created were sent by email to all active participants of the focus groups, i.e., at least participated in one focus group. The aim of this round of feedback was to identify whether the HS advisors had any suggestions for last improvements or adjustments regarding the roles and/or their descriptions and to investigate whether they have any feedback on the model. The final roles and descriptions were then presented and discussed with the project team.

## Results

### Phase 1: A narrative review of the literature

Fifty-five publications were identified and screened based on the primary researcher's experience in the field of implementation of the HPS approach. Of those 55 publications, 14 studies discussed the tasks of an HS advisor during the implementation process of the HPS approach in a school. Appendix I provides an overview of the included studies. Extracting the information created a list of 41 tasks of the HS advisor (Table 1, left side, if multiple references are cited, this task was mentioned in multiple studies). Grouping the tasks led to the identification of four main roles (Table 1, right side): (1) linking pin, (2) expert in the field, (3) involved adapter, and (4) critical friend. The two national coordinators of the HS advisors identified a fifth main role during the discussion of the findings: ambassador of the HPS approach (Table 1, right side). Based on the grouped tasks, the five roles were described as follows.

**Table 1:** From tasks to identified roles of an HS advisor: A narrative review.

**Tasks of an HS advisor, retrieved from scientific literature The HS advisor …**	**Identified roles of an HS advisor**
	
... is the link between the school and the external organizations and acts as the main point of contact (11, 19) ... approaches and connects possible external partners with the school (6, 11, 19) ... seeks the balance between top-down expertise and bottom-up involvement and convinces the school and external partners of the importance of this (19) ... helps to realize co-creation between all those involved (6) ... helps the school to formulate a shared vision between all stakeholders, including external partners (6, 21) ... connects the school with other schools so that experiences can be exchanged (18)	*Linking pin*
... familiarizes the school with the HPS approach (23)	*Expert in the field*
... helps to apply for (additional) financial resources (19) ... helps in conducting a needs assessment to determine the focus of the school (12, 19, 24) ... ensures to have up-to-date knowledge about possible evidence-based HP interventions and shares this with the school when necessary/possible (11, 13, 19) ... advises in the adoption, implementation and continuation of relevant HP interventions, taking into account the needs, wishes and possibilities of the school (6, 12, 21, 23, 25) ... advises on the ideal composition of the working group, e.g., teachers, parents, children, external partners (19) ... advises on an appropriate strategy to involve parents (19) ... shares experiences of best practices (19) ... assists the school in integrating the HP interventions within the core business (13, 24)	
... takes sufficient time to become (and remain) familiar with the school context and the dynamics in the school (19) ... invests in gaining the trust of the school (6) ... senses what the school needs, such as the type of guidance: e.g., active or mainly informative (19) ... recognizes momentum and uses it (6) ... has an overview of 'key players' in the school and the variety of roles the people have in the system (26) ... looks into physical and organizational structures that may promote effective implementation (18) .... is open to what is happening in the school and responds flexible to it (19) ... keeps an overview of what is happening in a school with regard to HP over time (18, 21) .... has an eye for the expectations of all those involved, their needs and wishes, and the available resources and possibilities of the school (11, 23, 27)	*Involved adapter*
... helps the school to understand that the implementation of the HPS approach cannot be achieved overnight and that patience is essential (13, 21)	*Critical friend*
... helps the school to understand that the implementation of the HPS approach is an on-going process of change and not a product to be done (13) ... ensures that sufficient time is taken in the preparations and that sufficient attention is paid to create support of all those involved (6, 19) ... is consciously creating constructive communication with the school (11, 25) ... advises the school not only about the content, but also about the process and cooperation (10, 13) ... helps the school to properly divide all tasks and to seek support when necessary (19) ... helps the school to deal with barriers and to find (creative) solutions (28) ... helps the school to understand the importance of feedback loops and to generate these, so that they can provide insight into the wishes and needs of those involved and can immediately adapt when activities or processes are running sub-optimal (19, 29) ... helps the school with regard to monitoring and evaluation of the process and impact, whereby the HS-advisor ensures that the evaluation has a broad focus, a distinction is made between short and long term goals, and the impact is evaluated by both effect evaluation (effect sizes) and context evaluation (how, for whom, where, under what circumstances) (6, 10, 19)	
... monitors that the focus and commitment do not disappear during the process (18) ... monitors the extent to which the activities actually seep into the context, i.e., become part of the DNA of the school, by looking at the extent and intensity of the activities and the coherence between the activities (21, 26) ... helps the school to think about sustainability, so that both the activities and vision of the HPS approach remain intact, even when personnel changes (18, 21)	
... helps the school to see the need to get started with the HPS approach (11) ... makes and keeps the school enthusiastic about the HPS approach (18, 21) ... helps the school to understand that tackling complex problems (such as obesity) also requires complex system-wide soluions and that a quick-fix mentality that focuses on *ad-hoc* interventions with a low intensity will not lead to structural change (19, 21) ... helps the school to receive support from the school board, by involving this board and pointing out their role in this (19) ... encourages possible external partners to contribute to the implementation of the HPS approach in the school (21)	*Ambassador of the HPS approach*

#### Linking pin

The HS advisor links the school to external partners and helps to enhance the participation of all stakeholders in the school. The HS advisor helps to create a co-creation process in which all partners collaborate, and a balance is found between the expertise of external partners and the involvement of the stakeholders in the school.

#### Expert in the field

The HS advisor has the expertise and up-to-date knowledge about specific health topics, such as physical activity, smoking, and wellbeing, and the HPS approach, in general. The HS advisor uses this knowledge, together with his/her own experiences, to support the schools with the implementation and continuation of the HPS approach.

#### Involved adapter

The HS advisor is involved, observes the specific context and dynamics in the school, and has a close collaboration with the HS coordinator. This helps the HS advisor to gain insight into the wishes, needs, expectations, and opportunities of the school, and he/she can deal with this in a flexible manner.

#### Critical friend

The HS advisor is a “detached outsider” of the school. He/she guides the school in the implementation, continuation, and evaluation of the HPS approach. The HS advisor not only supports, motivates, and advises the school but also remains critical during the whole process.

#### Ambassador of the HPS approach

The HS advisor creates enthusiasm for the HPS approach in the school and at external organizations by making them aware of the importance and the need to work with such an integral approach.

### Phase 2: Individual interviews with HS advisors

The five roles resulting from phase 1 were discussed with 12 HS advisors from eight public health service regions^2^ throughout the Netherlands during individual interviews (NB conducted six interviews; BvD conducted five interviews of which one interview was conducted with two HS advisors together). Participants (all females) underlined that each public health service had a different organizational structure for the HS advisor role, with all respondents also having other responsibilities next to their HS advisor role. Most were provided with hours from their organization and/or from municipal funds, sometimes supplemented for specific schools with the 10 h of advice a school receives from the national program. A few HS advisors were fully dependent on these 10 h for their advisory roles, which gave them less flexibility. All HS advisors worked in a team with other advisors, but the sizes of the team varied between three and 12 people. The participating HS advisors differed regarding working experience as HS advisor: Eight HS advisors had more than 10 years of working experience, one HS advisor had 4–10 years of working experience, and three HS advisors had 1–3 years of working experience. On average, the interviews lasted 83 min (ranging between 64 and 105 min).

Findings showed that all participants recognized the five identified roles in their own work as HS advisor and the work of HS advisors in general. According to the HS advisors, the various roles describe a complete overview of their work, and no roles were missing. However, it was also mentioned that applying all these roles can be challenging, because it depends on the situation as to which role(s), ore part of a role, are applicable.

“*All roles are recognizable to me. They provide a complete overview of my work as HS advisor. I also do not miss a specific role. However, the extent to which I apply a role can differ quite a lot per school and situation.”* (HS advisor, 4–10 years of working experience).

Several nuances were also given for each specific role as follows.

#### Linking pin

Most participants mentioned that this role is the most recognizable one. It describes very specifically how you can support the schools when applying for this role. Since the HS advisor often actively works with local partners in a municipality, he/she can really have added value by linking the school to external partners, such as sports clubs or welfare organizations. These partners can, for example, help the school to promote a healthy lifestyle and wellbeing on a specific topic. Moreover, the findings showed that when discussing this linking pin role, the participants mainly focused on the external partners and hardly on the participation of the stakeholders in the school. Enhancing this participation was seen as mainly a task for the HS coordinator who operates within the school.

“*Yes, I recognize this role, but in my opinion the HS coordinator is the real linking pin inside the school.”* (HS advisor, >10 years of working experience).

#### Expert in the field

The participants perceived this role as important but challenging. They indicated to have knowledge and expertise about the HS program in general and when needed, they know how to find in-depth information about a specific health topic. However, since new insights or newly developed interventions constantly lead to developments in different topics, they find it challenging to keep their knowledge up to date. Several participants indicated that they have divided this task of having up-to-date knowledge among colleagues who also work as an HS advisor and that they consult each other when they need more in-depth information on a specific topic. In contrast, some participants mentioned that this was not possible as they did not have enough colleagues in their region who also work as an HS advisor. In general, many indicated that the support in this from the national HS program could be optimized.

“*I have the expertise and knowledge, but I do not think it is realistic to be an expert in everything. I know where to find the information when needed. Though, in my opinion a general and up-to-date overview per theme is missing. I have to be very proactive to find all information*.” (HS advisor, 1–3 years of working experience).

#### Involved adapter

The perceptions regarding this role varied considerably among the participants. Some perceived it as the most crucial role, and others perceived it as the least recognizable one.

“*This is very much recognizable. I think it is the main role of an HS advisor*.” (HS advisor, 1–3 years of working experience).

“*I do not recognize this role as much as the other roles. Schools know very well what they want, we do not have to take them by the hand. The school is in the lead, and when there are any questions, they can let me know.”* (HS advisor, >10 years of working experience).

Despite varying perceptions, HS advisors agreed that collaboration with the HS coordinator, who is specifically mentioned within this role, is very important as he/she knows the school best. This HS coordinator can, therefore, help the HS advisor to gain more in-depth insight into the school context and its dynamics; the HS advisor can then support this HS coordinator in finding ways how to adapt HP actions and activities to that specific situation.

#### Critical friend

The word “critical” in this role triggered some participants. The more experienced HS advisors mentioned that being critical of the process and the decision for specific actions and/or activities is important to create effective and sustainable change. According to them, being critical can be seen as the added value of the HS advisor to the process, and he/she is a quality gatekeeper of the integral approach and the implementation of HP actions and activities. However, the HS advisors with less working experience perceived the word “critical” more often as negative and mentioned that they were very careful to apply this part of the role. In their perception, being critical could damage the good relationship with the school, which could lead to losing the school's support and enthusiasm.

“*The role is recognizable but being critical can be very challenging. You must find a balance in how critical you can be. You do not want to lose them. So, there is not always the luxury of being critical*.” *(*HS advisor, 1–3 years of working experience).

“*This role, including the part of being critical, should be more prominent in the work of HS advisor compared to what we do now. As an HS advisor you have specific expertise, and you can trust on this and stand for it. When you perceive resistance in the school, it is important to figure out the reason for this resistance.” (*HS advisor, >10 years of working experience).

#### Ambassador of the HPS approach

The findings showed that the participants, when discussing this ambassador's role, mainly talked about the adoption phase in the implementation process or when a school decides to start working with the HS program. They also indicated that in this adoption phase, it can be challenging to take this role due to how the Dutch HS program works. A school often has already received funding and decided on a topic, before they come into contact with an HS advisor. The ambassador's role is less needed, according to the participants. Also, it was indicated that when a school does not have the motivation to work on health promotion, they do not want to put their limited time into taking the ambassador's role to convince the school to continue with the HPS approach. They rather use that time to support other, more motivated, schools. Finally, the participants mentioned that in this ambassador role, it is important not to forget the potential external partners, as they can be very valuable in supporting a school in promoting healthy lifestyle and wellbeing.

“*This role is obvious and a natural part of the HS advisor. However, it is not always easy to apply this role due to a lack of time.” (*HS advisor, >10 years of working experience).

“*A school is often quite busy with many other issues; they just do not want to focus on health promotion then as well. I want to give them this space, I do not want to ruin the relationship, by pushing them all the time.” (*HS advisor, >10 years of working experience).

### Phase 3: Focus groups with HS advisors

The five roles and their descriptions were improved after phase 2 and then used in phase 3: the focus groups. In total, 16 HS advisors subscribed to be interested to participate of which 13 HS advisors have participated in one or more focus groups. About half of these participants (*n* = 7) had also participated in the individual interviews. The 13 participants (all females) were located in 11 different regions in the Netherlands (see note 2), with some participants also having worked in other regions than their current location (17). Regarding working experience, the group was quite diverse: four HS advisors had 1–3 years of work experience, three HS advisors had 4–10 years of working experience, and six HS advisors had more than 10 years of working experience.

Ten participants joined in the June session (six HS advisors in sub-session 1a and four in sub-session 1b) and nine participants joined in the September session (four HS advisors in sub-session 2a and five in sub-session 2b). Each sub-session lasted for 2 h. Minor improvements to the description of the roles were suggested in the June session, such as that the linking pin role could be a bit more described in relation to the HS coordinator of the school. The September session, in which the roles had to be applied in a case, resulted in an important insight into the role of the involved adapter. It was observed that the participants were struggling with it. More specifically, they indicated that the title of this role was confusing to them. At the same time, however, they emphasized that they felt this role was relevant in every situation they encountered as an HS advisor. They mentioned that an HS advisor is always searching or navigating for the best fit with the specific school context, based on the close contact they maintain with the school through an HS coordinator (who in the Dutch context is a school employee). According to them, this role should, therefore, be stated on a more central level than the other ones.

“*I wonder whether you should call it involved adapter, aren't you just an adapter and being involved is part of that? I do really believe that this role is at the base of the work of an HS advisor; it then depends on the support that is needed which other roles you will take.” (*HS advisor, >10 years of working experience).

Renaming this role “navigator” was proposed as a suitable alternative because, in the Dutch context, an HS advisor is essentially someone who guides, coaches, supports, and empowers the school to become a healthy school, or in other words, helps the school navigate their unique context via the good relationship they build with a central contact person in the school.

### Phase 4: Final check of the key roles

After phase 3, the roles were finalized by the two principal researchers, and a model was created to visualize how they relate to each other. Both the description and the model were presented to all active focus group participants (*n* = 13), and they were asked for final suggestions. Seven participants were available to respond. None of the respondents had any final adjustments, additions, or other comments and agreed to the final description and model of the five roles of the HS advisor in Dutch schools. Likewise, no improvements were suggested in the project team when discussing the final roles.

“*A very nice overview of the work of an HS advisor.” (*HS advisor, >10 years of working experience).

“*A good representation of the discussions we had, my compliments for that.” (*HS advisor, 4–10 years of working experience).

“*Cool new name [*read: for the involved adapter role*], definitely an improvement to change from involved adapter to navigator. This name fits much better.” (*HS advisor, 4–10 years of working experience).

**Figure 2:**
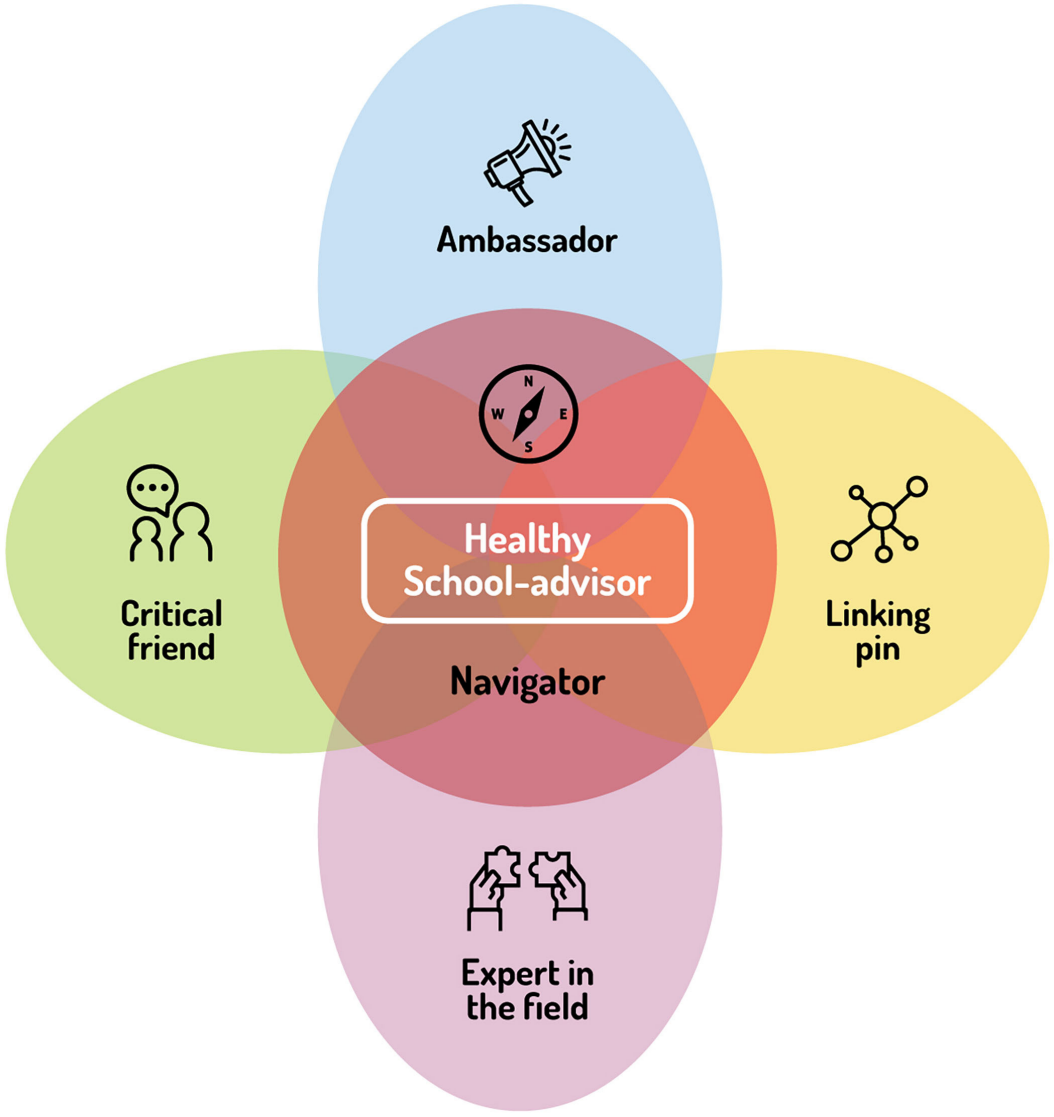
The five roles of a Healthy School-advisor.

The final description of the five roles is presented in Box 1 and is shown in Figure 2.

Box 1 **Description of the five roles of an HS advisor based on the Dutch context.**

Central role:

**Navigator:** The HS advisor has close contact with the HS coordinator. Due to this contact, the HS advisor has insight in the specific context and dynamics in the school and has insight in the wishes, needs, expectations, and opportunities of the school. He/She can deal with this in a flexible manner.

Other roles:

**Linking pin:** The HS advisor links the school to external partners, such as the municipality, and helps the HS coordinator to enhance the participation of the stakeholders in the school. The HS advisor helps to create a co-creation process in which all partners collaborate and a balance is found between the external expertise and opportunities and the involvement of the stakeholders in the school.

**Expert in the field:** The HS advisor has expertise and up-to-date knowledge about the HPS approach and the local opportunities. When needed, he/she is able to gain in-depth knowledge about a specific topic from colleagues or theme-related institutes. The HS advisor uses this knowledge, together with his/her own experiences, to support the schools with the implementation of the HPS approach.

**Critical friend:** The HS advisor is a ‘detached outsider' of the school. He/She guides the school in the implementation of the HPS approach. The HS advisor supports, motivates, and advises the school and is a gatekeeper of the quality: He/She follows the whole process critically and helps the school adjust the course of the process when needed.

**Ambassador of the HPS approach:** The HS advisor creates enthusiasm for the HPS approach in the school and among external partners by making them aware of the importance and the need to work with such an integral approach. This is done in the beginning of the process to create enthusiasm to start with the HPS approach, but also during the process to keep the enthusiasm alive.

## Discussion

The current study identified and described the key roles of an HS advisor during the implementation process of the HPS approach in schools in the Netherlands. The study was based on a co-creation process involving Dutch HS advisors, as well as researchers and national representatives and coordinators of the HS program. The co-creation process consisted of four main phases: (1) a narrative review, (2) individual interviews with HS advisors, (3) focus groups with HS advisors, and (4) a final check of the roles. This process has led to the identification and description of five key roles of the HS advisor: a navigator role as a more central one, and four other roles, namely, linking pin, expert in the field, critical friend, and ambassador of the HPS approach. Despite the fact that many studies mention the importance of support from HS advisors to schools during the implementation process of the HPS approach, only limited research has been performed on specific roles of such advisors (18–21). Therefore, these five roles added to the studies of Boot et al. (9, 10) who described the work of an HS advisor as an effective change agent that convinces the school of the benefits of working on HP and guides the process of change. The current study has expanded their work to a complete description of the five key roles of HS advisors. The participants in the co-creation process all agreed that these five roles and their descriptions were recognizable and that it provides a complete overview of the work of an HS advisor in the Netherlands. This consensus is meaningful since the tasks of an HS advisor in the Netherlands can differ quite a lot depending on, e.g., the available budget and organizational structure of the regional public health services the HS advisor is employed by, the knowledge, skills, competencies, and personal preferences of the HS advisor, and the specific context of the school the HS advisor supports (6, 10, 19). In other words, it seems that an overarching level is reached by describing the work of an HS advisor by the five roles. This seems to make it applicable to all HS advisors in the Netherlands without inhibiting them to work in an adaptive and context-oriented manner. Nevertheless, it is important to keep in mind that the application of the roles depends on the specific context of when and how each role can best be fulfilled. This means that the HS advisor should continuously navigate in that context to provide the support that optimally fits the specific situation. Therefore, the navigator role was placed on a more central level in the model (Figure 2). The other four roles may be relevant to a greater or lesser extent in a specific situation or at a particular stage in the implementation process. In practice, the roles are all closely linked to each other, so some overlap is, therefore, inevitable.

Even though the five roles seem to provide a clear overview of the work of the HS advisor that is generalizable for the Dutch context, it does not mean that it is applicable in other countries. This was also not the aim of the study and was even perceived as impossible since each national context is different, and every country has its own system with specific contextual factors regarding the implementation (support) of the HPS approach in schools (20, 22). The identified and described five roles may, however, guide and inspire HS advisors from other countries to contextualize the roles to their specific national context. This may help to create or optimize sufficient support for schools during the implementation process of the HPS approach. Even though not every country may have a system of HS advisors, these roles can be a starting point for stakeholders in and around schools on how schools can best be supported. In addition, the participatory research approach we used, in which we utilized a co-creation process comprised of four phases to identify and describe the five roles, may serve as a guide or inspiration for other researchers both nationally and internationally.

Looking specifically at the perceptions of the HS advisors on the five roles during the interviews (phase 2) and focus groups (phase 3), it can be observed that some HS advisors perceived more difficulties with the roles than others. This is not surprising, as the roles describe how an HS advisor can optimally support schools. It can be very challenging though to figure out how to fulfill these five roles in different contexts. HS advisors indicated, for example that the navigator role (previously named involved adaptor) was a bit vague and the least recognizable role for them, while others described this role as the most crucial one. According to them, navigation should be part of every situation to be able to work in a context-oriented manner. Also regarding the critical friend role, some issues were discussed. Some HS advisors perceived that being critical when supporting schools is challenging in real life. Others had no difficulty with it and thought that being critical was the added value of an HS advisor. They identified themselves as quality gatekeeper who has specific expertise to add to the process. As this study is part of an overarching research project to develop training for HS advisors, we aim to include the roles in this training. In this way, HS advisors can discuss the five roles based on real-life working situations and practice translating the roles into specific support they provide to each school. Even though the practical application of different roles was not the main focus of this study, an interesting finding regarding the ambassador's role was observed throughout the phases of the current study. The ambassador's role was specifically added by the national coordinators as something they expected HS advisors to do. Although HS advisors involved in this study agreed with this, they also noted that in practice taking on this role was sometimes difficult to achieve or not needed anymore. In their perception, the role matters most in the adoption phase of the implementation process, when schools choose to start working with the HS program and prioritize a specific health topic. HS advisors experienced that they were often contacted by the school after the decision was made to, indeed, work with the HS program and on which health topic they wanted to focus. According to HS advisors, this results in mainly supporting schools that are already motivated and often only need a little push to implement HP in the school. However, the ambassador's role is still important in these schools to keep all stakeholders in the school motivated and engaged throughout the whole implementation process to create a sustainable impact. In addition to this, the ambassador's role is also important to proactively convince the other, maybe less motivated, schools to start working with the HS program. Considering these two aspects, it can be recommended to pay specific attention to the broad application of the ambassador's role when discussing or applying for the roles.

### Strengths and limitations

Several strengths and limitations should be considered when discussing the results of the current study. An important strength of the study was that both researchers and professionals in the field were involved in co-creation processes enabled by participatory research. This led to findings that were shaped by the insights from science and experiences from practice. In addition, the methods were based on a combination of research and practice. The narrative review provided the existing knowledge and insights from the scientific literature, which served as input for the interviews and co-creation sessions with the HS advisors. Their experiences and perceptions from “the field” led to the further identification and description of the five roles and made sure that these fit real-life practice. The study also had several limitations. Since the narrative review was not conducted systematically, some relevant studies may have been missed. However, almost no studies focused on the tasks of an HS advisor in particular. Instead most studies focused on factors important for the implementation of the HPS approach in which the task of an HS advisor was often only mentioned in one or two sentences in the discussion section of an article. Therefore, it is unlikely that a systematic review would have led to more relevant articles. Another limitation was the limited number of HS advisors included in the interviews. Since only 12 HS advisors were interviewed, with all having their own context, it is hard to say whether data saturation was reached. However, comparable responses were given to the roles, suggesting that data saturation was reached. This, together with the fact that different roles would be discussed again in the focus groups, led to the decision to continue with the process after interviewing these 12 HS advisors. Finally, even though many efforts were taken, not all regions in the Netherlands were represented in the interviews and focus groups. This could have led to the findings that are not completely representative of all HS advisors in the Netherlands. However, the participating HS advisors came from regions all over the Netherlands, and no more than two HS advisors from the same region were involved. Since full consensus was reached in phase 4 of the study among not only the participating HS advisors but also the national representatives and coordinators of the Dutch HS program, generalizability of the findings does not seem to be an issue.

## Conclusion

The current study has led to the identification and description of five roles of HS advisors in their support of schools during the implementation process of the HPS approach. The five roles are: (1) navigator, (2) linking pin, (3) expert in the field, (4) critical friend, and (5) ambassador of the HPS approach. The navigator role is hereby centrally stated because navigating is needed in every situation to be able to work in a context-oriented manner. The application of the other four roles is depended on the specific situation, and these can be seen as a playground in which context-oriented support takes place. Overall, the five roles can provide guidance to all HS advisors on what is needed to provide sufficient and context-oriented support to schools. Moreover, the roles may inspire and guide professionals from other countries from science and practice who are involved in implementing the HPS approach to adapt the five roles to their own national context, and thereby optimize the implementation support of the HPS approach provided to schools in their country.

## Data availability statement

The raw data supporting the conclusions of this article will be made available by the authors, without undue reservation.

## Ethics statement

The studies involving human participants were reviewed and approved by Ethics Review Committe Health, Medicine and Life Sciences (FHML-REC) of Maastricht University. The patients/participants provided their written informed consent to participate in this study.

## Author contributions

NB and BvD acted as principal researchers. NB drafted the manuscript. BvD provided continuous input. All other authors provided advice on the study, the analysis, and the results, as well as provided feedback on the manuscript. All authors were involved in the design of the study and read and approved the final manuscript.

## Footnotes

^1^Note that each Public Health Service has the autonomy to set-up its own organizational structure to finance and further specify the responsibilities of the HS advisor. Almost all HS advisors also have a broader health promotion task in multiple municipalities and the size of the team HS advisor can differ per organization, depending on the choices the Public Health Service makes. Therefore, it can differ how much time and flexibility the HS advisor has with three main finance structures for this role (or a combination): the previously mentioned 10 h of advice funded from the national program, municipal funds if a municipality decides to buy additional hours or flexibility in provided time funded by the regional Public Health Service themselves.

^2^There are 25 Public Health Services in the Netherlands, each servicing multiple municipalities.
