# Supplementary material for: Supporting schools during the implementation of the health-promoting school approach: The roles of a healthy school advisor
Source: Front Public Health. 2022 Dec 15;10:960873. doi: 10.3389/fpubh.2022.960873 (PMC9797735; doi:10.3389/fpubh.2022.960873)
Supplement: Supplementary file 1 [file Table_1.PDF]

Appendix I – overview of studies included in the narrative review phase

Ref. nr.	Author and year	Country of research	Title of article/dissertation
6	Bartelink, N. (2019)	Netherlands	Evaluating Health Promotion in Complex Adaptive School Systems: The Healthy Primary School of the Future [Dissertation]
9	Boot, N. (2011)	Netherlands	Health promotion and secondary education: marriage of convenience of real love? A study about the implementation of school health policy in secondary schools [Dissertation]
10	Boot, N., et al. (2012)	Netherlands	Implementation of School Health Promotion: Consequences for Professional Assistance.
12	Leurs, M. (2008)	Netherlands	A Collaborative Approach to Tailored Whole-School Health Promotion [Dissertation]
13	Pucher, K.K. (2015)	Netherlands	Optimizing Intersectoral Collaboration in School Health Promotion: Creating Win-Win Situations and a Systematic Implementation Based on the Diagnosis of Sustainable Collaboration Model [Dissertation]
18	Rowling, L. (2011)	N.A.[1]	Filling the Black Box of Implementation for Health-Promoting Schools
19	Verjans-Janssen, S.R. (2020)	Netherlands	Implementation and Evaluation of a Context-Based Physical Activity and Nutrition Intervention in the Primary School Environment [Dissertation]
21	Deschesnes, M., et al. (2003)	N.A.	Comprehensive Approaches to School Health Promotion: How to Achieve Broader Implementation?
23	McIsaac, J.D., et al. (2016)	Austria, Norway, UK, Canada, US, Australia, New Zeeland & Western Pacific	Interventions to Support System-Level Implementation of Health Promoting Schools: A Scoping Review
24	Langford R., et al. (2015)	US, Australia, West-European countries, Mexico, India, China, Canada, Tanzania, Egypt	The World Health Organization's Health Promoting Schools Framework: A Cochrane Systematic
25	Bos, V., et al. (2010)	Netherlands	Health promotion and prevention in education: current status, effectiveness and experiences from Regional Health Services and schools [national evaluation report]
26	Hawe, P., et al. (2009)	N.A.	Theorising Interventions as Events in Systems.
27	Darlington, E., et al. (2018)	France	Implementation of Health Promotion Programmes in Schools: An Approach to Understand the Influence of Contextual Factors on the Process?
28	Ter Haar, W. (2014)	Netherlands	Communicating and improvising: dealing with dynamics and complexity when developing

			and implementing health interventions [Dissertation]
29	Snyder, S. (2013)	N.A.	The Simple, the Complicated, and the Complex: Educational Reform through the Lens of Complexity Theory

1: Papers indicated with not applicable are discussion papers relating to implementation that are not focused on a specific country
